# Are current follow-up intervals justified in patients with non-emergent aortic surgeries?

**DOI:** 10.1093/icvts/ivae226

**Published:** 2024-12-28

**Authors:** Joseph Kletzer, Tim Berger, Stoyan Kondov, Thomas Bleile, Aleksandar Dimov, Victoria Werdecker, Martin Czerny, Bartosz Rylski, Maximilian Kreibich

**Affiliations:** Department of Cardiovascular Surgery, University Hospital Freiburg Heart Centre, Freiburg, Germany; Faculty of Medicine, University of Freiburg, Freiburg, Germany; Department of Cardiovascular Surgery, University Hospital Freiburg Heart Centre, Freiburg, Germany; Faculty of Medicine, University of Freiburg, Freiburg, Germany; Department of Cardiovascular Surgery, University Hospital Freiburg Heart Centre, Freiburg, Germany; Faculty of Medicine, University of Freiburg, Freiburg, Germany; Department of Cardiovascular Surgery, University Hospital Freiburg Heart Centre, Freiburg, Germany; Faculty of Medicine, University of Freiburg, Freiburg, Germany; Department of Cardiovascular Surgery, University Hospital Freiburg Heart Centre, Freiburg, Germany; Faculty of Medicine, University of Freiburg, Freiburg, Germany; Department of Cardiovascular Surgery, University Hospital Freiburg Heart Centre, Freiburg, Germany; Faculty of Medicine, University of Freiburg, Freiburg, Germany; Department of Cardiovascular Surgery, University Hospital Freiburg Heart Centre, Freiburg, Germany; Faculty of Medicine, University of Freiburg, Freiburg, Germany; Department of Cardiovascular Surgery, Robert Bosch Hospital, Stuttgart, Germany; Department of Cardiovascular Surgery, University Hospital Freiburg Heart Centre, Freiburg, Germany; Faculty of Medicine, University of Freiburg, Freiburg, Germany

**Keywords:** TEVAR, EVAR, open surgery, follow-up, aortic events, complications, surveillance

## Abstract

**OBJECTIVES:**

Evidence for different surveillance protocols following aortic treatment is still lacking. The aim of this study was to analyse the clinical relevance of a first follow-up visit after 6 months.

**METHODS:**

Between January 2018 and December 2019, 464 patients treated for non-emergent aortic pathologies were retrospectively analysed. The incidence of aortic events during follow-up (ie, death, reintervention, endoleaks, anastomotic/new aneurysms and diameter progression over time) was investigated. A discrete-time non-homogeneous Markov Chain Model was used to analyse the data and to arrive at the number of skipped 6-month follow-up visits needed to harm a patient.

**RESULTS:**

After 6 months, 2 (1.77%) patients had died, 15 (15.31%) patients suffered from aortic events and a total of 4 (3.67%) patients had undergone reintervention after endovascular surgery, compared to 0 deaths, 2 (0.59%) patients with aortic events and 5 (1.48%) reinterventions after open surgery. In our Markov Chain Model, after 6 months, 4.75% of patients showed aortic events, received a reintervention or died. Sixty patients would need to skip their 6-month follow-up visit for one indication for reintervention to go unnoticed. Only 24 would need to skip it for one complication to go by unnoticed. This number is 55 after open surgery and 9 after endovascular surgery.

**CONCLUSIONS:**

After elective endovascular or open aortic surgery without immediate in-hospital postoperative aortic events, the first follow-up visit after 6 months is important. Extending the first interval to longer time periods might lead to a considerable increase in missed aortic events. The cost and radiation exposure of frequent follow-ups must be balanced against the benefits of early preventative aortic interventions, warranting further research.

## GRAPHICAL ABSTRACT

**Figure:**
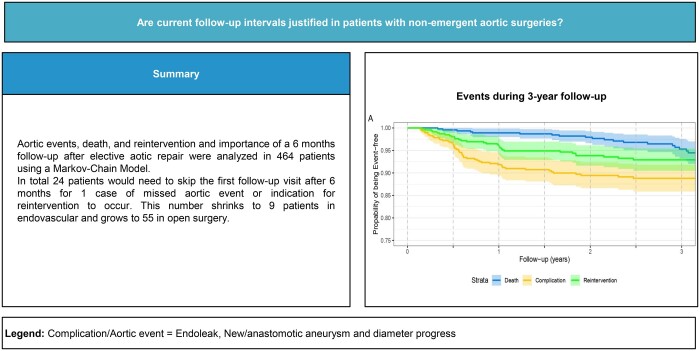

## INTRODUCTION

A close follow-up following aortic treatment because of long-term aortic events such as various kinds of endoleak, generation of new aneurysms such as anastomotic aneurysms or infection is regularly at the centre of research, as they might lead to more severe adverse events as the pathology progresses [1, 2]. Regular postoperative clinical follow-up is paramount to detect and avoid these aortic events. The American College of Cardiology/American Heart Association (ACC/AHA) 2022 aortic guidelines suggest that 1–7% [3] of openly repaired aortic aneurysms may require reintervention due to treatment failure during follow-up. Other work suggests reintervention rates of 6–25% after thoracic endovascular aortic repair (TEVAR) or frozen elephant trunk treatment [4, 5].

Nevertheless, current recommendations lack evidence for optimizing follow-up intervals, as they are mostly formed by data generated as a by-product in larger trials, which were initially powered for completely different end-points [6]. This study aims to investigate current surveillance protocols after aortic surgeries and shed light on optimal follow-up intervals in patients who underwent elective, non-emergent aortic surgery, either open or endovascular.

## PATIENTS AND METHODS

### Ethics statement

IRB approval was obtained on 04 February 2021 (No. 20-1302) by the institutional review board of the University of Freiburg, and the need for individual informed consent was waived due to retrospective analysis.

### Patients

**Figure 1:**
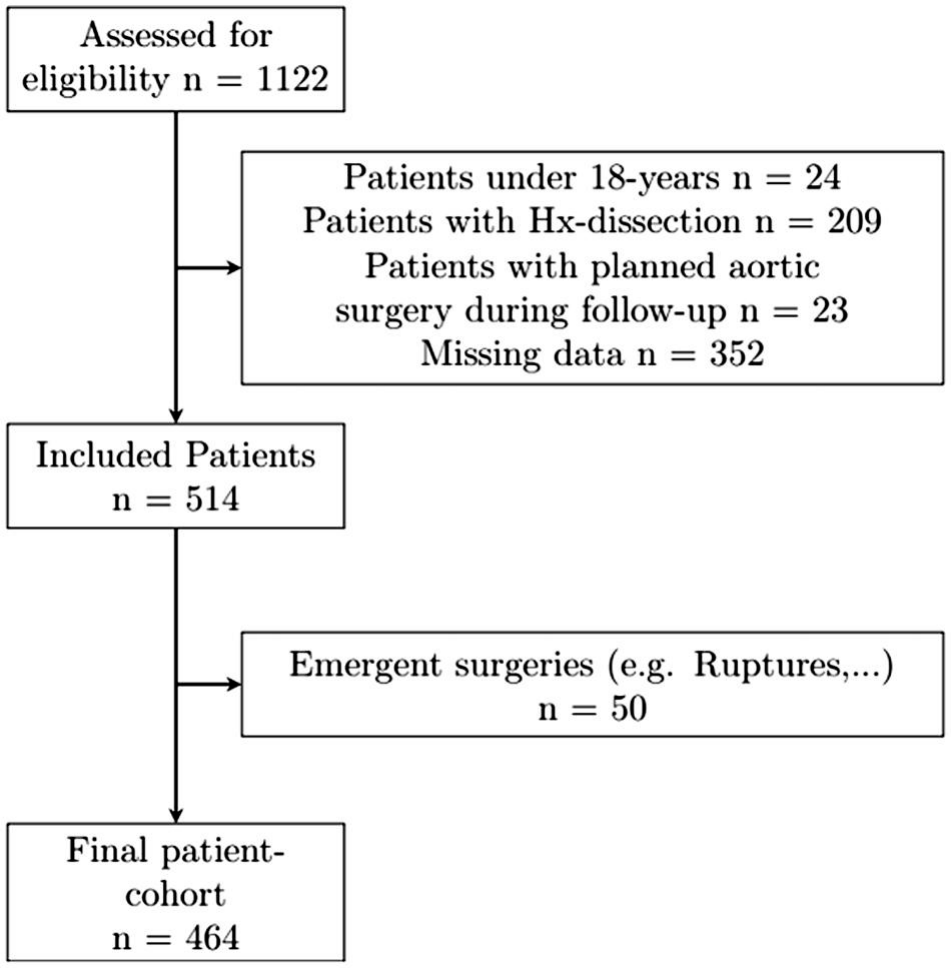
Flow chart of patient exclusion in this study. *n*: number of patients; Hx: history of.

This study analysed patients who were postoperatively discharged following treatment for elective, non-emergent aortic disease between January 2018 and December 2019 at the University Hospital Freiburg—Heart Centre. We included patients treated for thoracic or abdominal aortic aneurysm, Leriche syndrome and penetrating aortic ulcer (PAU). Patients with in-hospital aortic events following the index procedure or patients who did not participate in regular follow-up visits in our aortic clinic were not included. In addition, we excluded patients younger than 18 years, patients with a history of dissection, patients with planned further surgery of the aorta, as well as emergent aortic surgery such as ruptures. Patients were also excluded if there was no or only one follow-up visit with corresponding computed tomography angiography (CTA) imaging available. This process is visualized in Fig. 1.

### Data collection and outcome definitions

Data were taken from our centre’s dedicated aortic database. CTA findings were interpreted and validated by radiologist physicians. They were then manually taken from our electronical patient database for a detailed assessment of possible aortic events. As primary outcomes, we defined critical events that would signify a serious impairment of our patient’s health:

1. Aortic events (defined in secondary outcomes)
2. Reintervention (open or endovascular)
3. Any cause of death

Secondary outcomes were defined as each item of aortic events:

1. New type I-IV endoleak
2. Diameter progression ≥ 1 cm [7] of residual aneurysm
3. Formation of a new aneurysm (eg, Anastomotic false aneurysm)

Additionally, in the case of endovascular surgery, endoleak Ia, Ib and II were assessed intraoperatively using fluoroscopy. As a baseline reference, immediate postoperative imaging and surgical reports were used. Surveillance CTA scans were obtained at follow-up visits, which were scheduled as per clinical standard, current EACTS/STS guidelines, patient compliance and clinical condition. Our internal standards dictate a first follow-up visit after 6 months in all aortic cases. As per surgeon preference, based on intraoperative conditions, an earlier visit was scheduled in some patients. All patients underwent either:

- Open surgery (affecting the aortic root, aortic arch, ascending or abdominal aorta)
- Endovascular treatment (TEVAR, EVAR)

All patients were treated for either thoracic or abdominal aortic pathology.

### Statistical analysis

Baseline characteristics were divided based on invasiveness of surgery, although we did not aim to directly compare open with endovascular surgery. This division was solely done for the sake of interpretation and the generalizability of our results. As patients with missing outcome variables were dropped before analysis, this was a complete case analysis regarding the first two follow-up visits. No imputation was performed for any other missing covariates. Mean (SD), number (percentage) or median [interquartile range (IQR)] was used to express data. Analysis was performed using IBM SPSS Statistics 27 for Windows (SPSS Inc.; Armonk, NY, USA) and RStudio (RStudio: Integrated Development for R. RStudio, Inc., Boston) using R Version (R Core Team (2023). R: A Language and Environment for Statistical Computing. R Foundation for Statistical Computing, Vienna, Austria). The Shapiro–Wilk test and Q-Q plots were used to assess normal distribution of data. Normally distributed data were then shown as mean (SD). Non-normally distributed data were presented as median (IQR) and compared using the Wilcoxon rank-sum test. Nominal data were displayed as a number (percentage) and compared using the Fisher exact test for an expected cell count of <5 and the *χ*^2^-test for the remaining data. Visualization of data was done using GraphPad Prism (version 9.0.0 for Windows, GraphPad Software, Boston, MA, USA) and with the ggplot2 [8], survival [9], survminer [10] and the diagram [11] packages for R.

**Figure 2:**
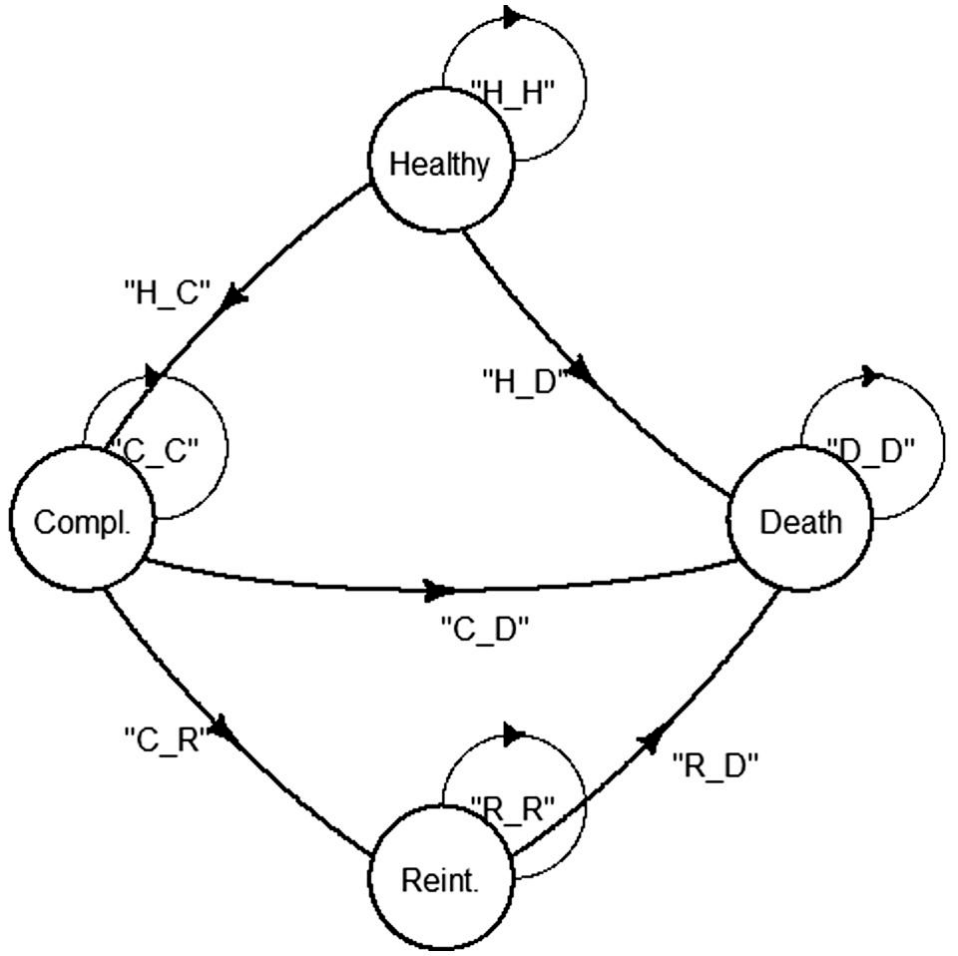
General illustration of the Markov Chain Model used in this study. H: healthy; C: complication; R: reintervention; D: death.

To assess the incidence of postoperative aortic events, we did Kaplan–Meier analyses for the outcome variables noted before. To model health outcomes over time based on available data, we decided to produce a discrete time non-homogeneous Markov Chain Model (MCM; Fig. 2). In this model, healthy patients were specifically defined as patients who did not show any aortic events as defined above, received aortic reintervention or had died. More details on the methods used can be found in the Supplemental Material.

## RESULTS

### Baseline patient characteristics and operative details

Patient baseline characteristics are summarized in Table 1. Overall, 351 (76%) of the 464 patients in this study were male. The median age of the cohort was 69 years (58–76). Three-hundred forty-eight patients (75%) were treated with open surgery. From these, 110 (32% of open surgery) patients had abdominal aortic pathology. Patients undergoing open surgery were younger than patients receiving endovascular treatment [median 77 years (69.5—80.5) vs 65 years (56.0—74.0); *P*-value < 0.001]. Remaining baseline characteristics can be found in the Supplemental Material.

**Table 1:** Baseline characteristics of the studies cohort

		Surgery		
Characteristic	**Overall**,^a^*N* = 464	**Endovascular**,^a^*N* = 116	**Open**,^a^*N* = 348	***P***-value^b^
Sex (male)	351 (76%)	94 (82%)	257 (74%)	0.079
Age (years)	69.00 (58.00, 76.00)	77.00 (69.50, 80.50)	65.00 (56.00, 74.00)	<0.001
Body mass index	26.35 (23.80, 29.23)	26.80 (23.80, 29.70)	26.20 (23.80, 29.10)	0.3
Diabetes mellitus	49 (11%)	20 (17%)	29 (8.3%)	0.006
Coronary heart disease	199 (43%)	73 (63%)	126 (36%)	<0.001
Dyslipidaemia	229 (49%)	76 (66%)	153 (44%)	<0.001
Dialysis	1 (0.2%)	1 (0.9%)	0 (0%)	0.2
History of Hypertension	350 (75%)	98 (85%)	252 (72%)	0.005
History of smoking	91 (20%)	21 (18%)	70 (20%)	0.7
Bicuspid aortic valve	79 (17%)	0 (0%)	79 (23%)	<0.001
Peripheral artery disease	70 (15%)	28 (24%)	42 (12%)	0.001
History of stroke	45 (9.7%)	13 (11%)	32 (9.2%)	0.5
History of COPD	44 (9.5%)	16 (14%)	28 (8.0%)	0.062
Connective tissue disease	8 (1.7%)	0 (0%)	8 (2.3%)	0.2
Previous cardiovascular surgery	149 (32%)	66 (57%)	83 (24%)	<0.001
Aneurysm	420 (91%)	94 (82%)	326 (93%)	<0.001
Leriche syndrome	11 (2.4%)	0 (0%)	11 (3.2%)	0.073
Penetrating aortic ulcer	34 (7.3%)	25 (22%)	9 (2.6%)	<0.001
Abdominal aortic pathology	209 (45%)	93 (81%)	116 (33%)	<0.001

COPD: chronic obstructive pulmonary disease; IQR: interquartile range.

a *n* (%); Median (IQR).

b Pearson’s Chi-squared test; Wilcoxon rank-sum test; Fisher’s exact test.

One-hundred sixteen patients (25%) received endovascular treatment. Most of these patients received endovascular repair of abdominal aneurysms (80%) and 22 (20%) for thoracic pathology. Operative details are summarized in Supplementary Table S7.

### Postoperative outcomes

During a median follow-up period of a median of 28.8 months (15.84—39.84), a total of 23 (5.0%) patients died, while 52 (11.0%) developed items of aortic events, and 33 (7.1%) needed aortic reintervention. Patients who underwent open surgery had a longer overall hospital stay than patients after endovascular surgery [open vs endovascular; median: 9.5 (6.0—14.0) vs 7.0 (2.0—9.0); *P*-value < 0.001] as well as a longer stay in the intensive care unit [open vs endovascular; median: 2 (1.0—4.0) vs 1 (1.0—2.0); *P*-value < 0.001]. A detailed description of postoperative outcomes can be found in Supplementary Fig. S8.

### Kaplan–Meier analysis

#### Overall cohort

At 6 months after surgery, there were 2 (0.44%) deaths, 17 (3.88%) cases of aortic events and 9 (2.01%) cases of reinterventions. These numbers grew to 6 (1.33%) deaths, 37 (9.09%) patients with aortic events and 18 (4.26%) patients who underwent reintervention at 1-year follow-up.

**Figure 3:**
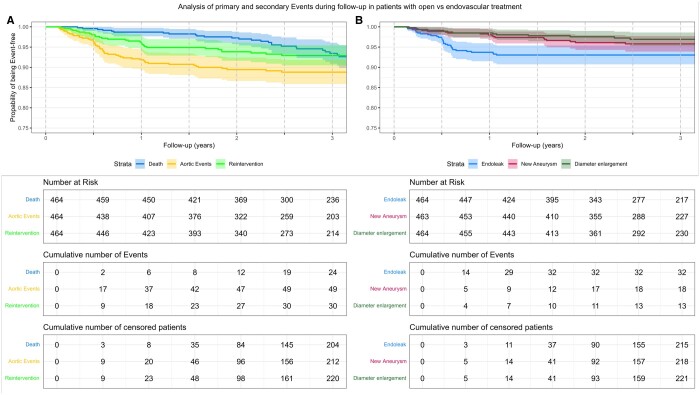
Kaplan–Meier curves of primary and secondary outcomes over 3 years of follow-up; lightly shaded area marks a 95% CI.

The items of aortic events included 14 (3.12%) cases of endoleak, 5 (1.1%) new aneurysms/anastomotic aneurysm and 4 (0.88%) cases of diameter progression were noted at 6-month follow-up. At 1 year, 29 (6.79%) patients had developed endoleak, 9 (2.04%) new aneurysms/anastomotic aneurysms were noted and 7 (1.58%) cases of diameter progression were found. A detailed account of numbers at risk, cumulative events, and Kaplan–Meier curves can be found in Fig. 3.

#### Endovascular vs open

At 6-month of follow-up, in patients who underwent endovascular aortic surgery, 2 (1.77%) patients had died, 15 (15.31%) patients suffered from aortic events and a total of 4 (3.67%) patients had undergone reintervention. Compared to 0 deaths, 2 (0.59%) patients with aortic events and 5 (1.48%) reinterventions in the open-surgery cohort.

One year after initial surgery, we noted 4 (3.66%) deaths, 29 (35.8%) patients with aortic events and 9 (9.09%) cases of reinterventions in the endovascular group, as well as 2 deaths (0.57%), 8 (2.45%) cases of aortic events and 9 (2.77%) reinterventions in open surgery.

Secondary item analysis of aortic events revealed the following: At 6-month follow-up, in patients who underwent endovascular surgery, 14 (13.73%) cases of endoleak, 3 (2.7%) cases of new aneurysm/anastomotic aneurysm and 3 (2.7%) cases of diameter progression were recorded. In the open-surgery group 2 (0.64%) new aneurysms, and 1 (0.32%) case of diameter progression occurred at 6 months after the initial surgery.

**Figure 4:**
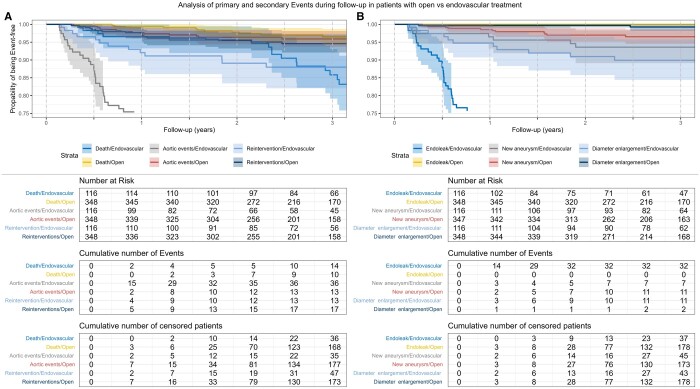
Kaplan–Meier curves of primary and secondary outcomes over 3 years of follow-up analysing patients with open and endovascular surgery; lightly shaded area marks a 95% CI.

After 1 year, the number of recorded endoleaks in the endovascular group was 29 (33.71%), new aneurysms/anastomotic aneurysms to 4 (3.77%), and diameter progression to 6 (5.83%). In contrast, after open surgery, there was no case of endoleak, 5 (1.49%) new aneurysms/anastomotic aneurysms, and 1 (0.29%) case of diameter progression. Cumulative patients at risk, events and censored patients, as well as Kaplan–Meier curves, can be found in Fig. 4. Respective illustrations and risk tables, comparing each outcome regarding endovascular vs open approach, can be found in Supplementary Figs S1–S6.

#### Thoracic vs abdominal

In the thoracic cohort, at 6 months, we recorded 0 deaths, 3 (1.22%) cases of aortic events and 2 (0.81%) reinterventions. At the abdominal level, there were 2 (0.97%) deaths, 14 (7.29%) cases of aortic events and 7 (3.54%) reinterventions. At 1 year, in patients after thoracic surgery, there was 1 (0.40%) death, 9 (3.81%) cases of aortic events and 3 (1.23%) interventions. There were 5 (2.48%) deaths, 28 (16.37%) aortic events and 15 (8.33%) reinterventions in abdominal surgeries. At 6 months, there were 3 (1.2%) new aneurysms/anastomotic aneurysms, 1 (0.39%) case of diameter progression and no case of endoleak detected in patients after thoracic surgery. After abdominal surgery, we detected 14 (7.17%) endoleaks, 2 (0.98%) new aneurysm/anastomotic aneurysms and 3 (1.47%) diameter enlargements. After 1 year, thoracic surgery patients showed 3 (1.21%) endoleaks, 4 (1.64%) new aneurysms/anastomotic aneurysms and 1 (0.40%) cases of diameter enlargement. Moreover, 26 (14.44%) cases of endoleak, 5 (2.53%) new aneurysms/anastomotic aneurysms and 6 (3.07%) diameter enlargements were found after abdominal surgery.

**Figure 5:**
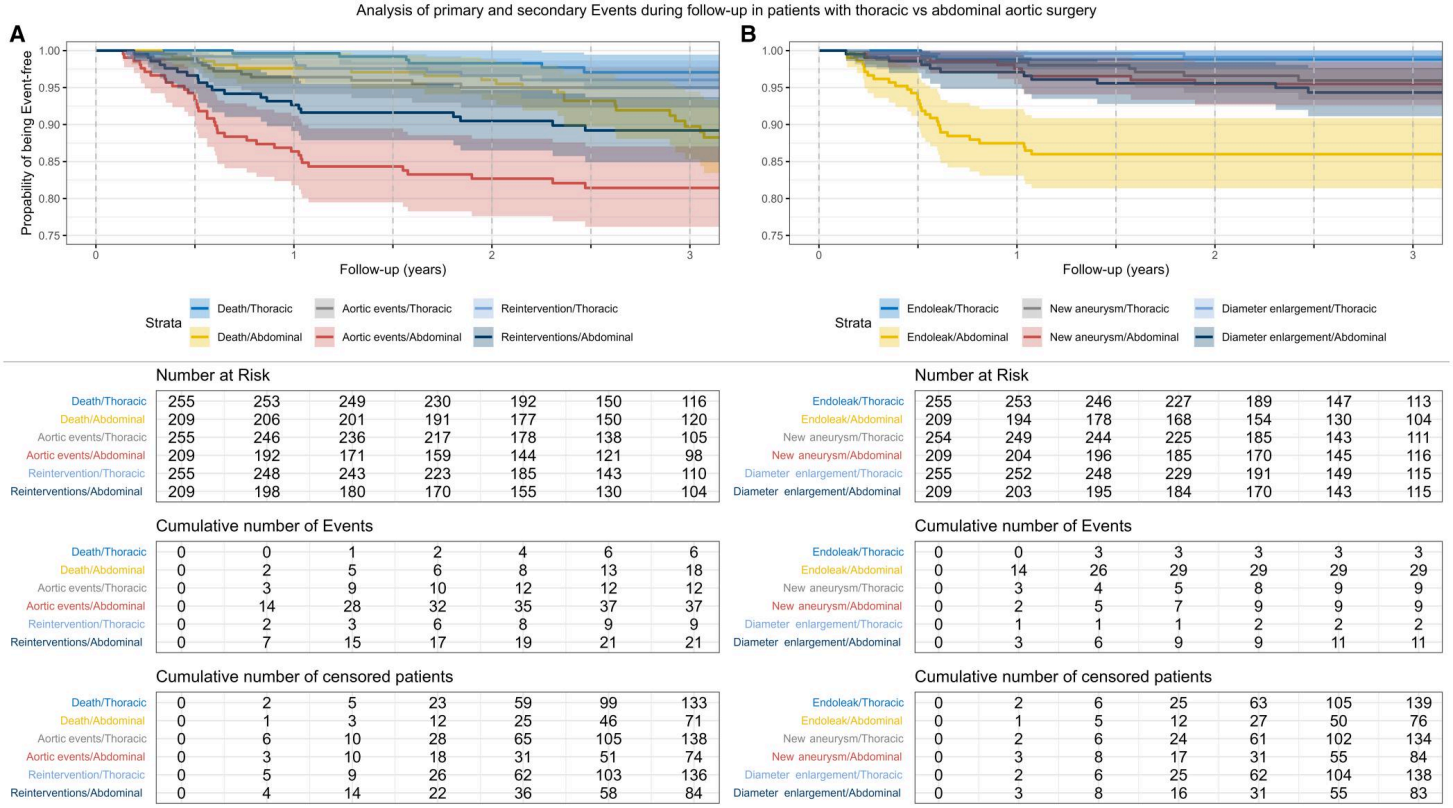
Kaplan–Meier curves of primary and secondary outcomes over 3 years of follow-up analysing thoracic vs abdominal surgery; lightly shaded area marks a 95% CI.

Figure 5 shows the number at risk, the cumulative number of events, the number of censored patients and the combined Kaplan–Meier curves of the data explained above. Supplementary Figures S7–S12 show Kaplan–Meier curves and risk tables for specific outcomes.

### Discrete time non-homogeneous Markov Chain Model

**Figure 6:**
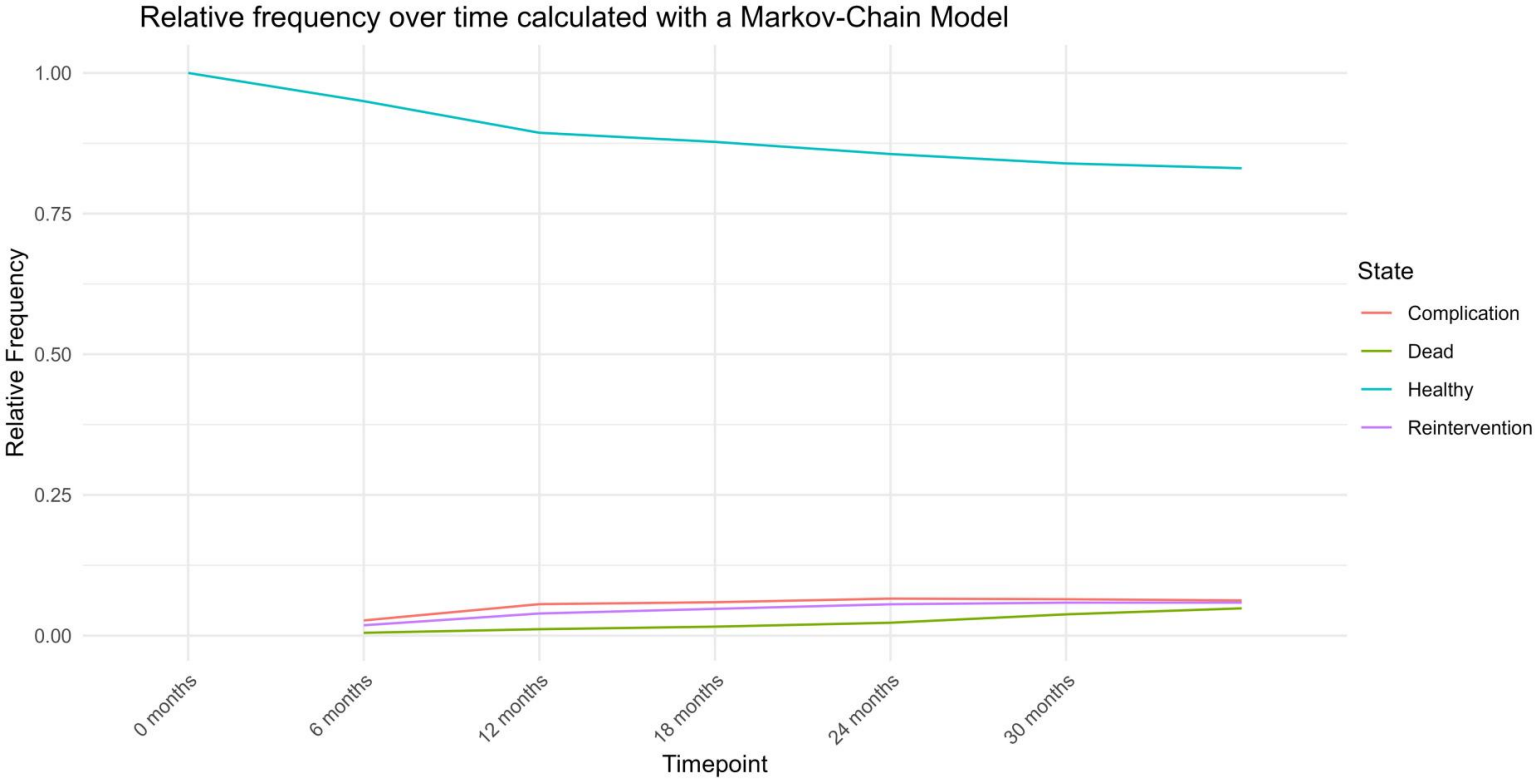
Discrete time non-homogeneous Markov chain simulation of a patient cohort *n* = 10 000.

Transition probabilities at each timestep can be found in Supplementary Tables S1–S6. A simulated patient cohort was illustrated in Fig. 6.

After 6 months of follow-up, there was a reduction in healthy patients by 4.75% (95.25% remaining). The relative frequency at the same time point was 2.58% for aortic events, 1.67% for reinterventions and 0.5% for death. At 12 months of follow-up, these relative frequencies increased to 5.78% aortic events, 3.62% reinterventions and 1.12% death, while only 89.48% patients remained healthy after surgery. At our last recorded timepoint, after 36 months of follow-up, relative frequencies reached 82.96% healthy patients, 6.49% aortic events, 5.68% reinterventions and 4.87% deaths. The number needed to harm (NNH) for missed complication or indication for reintervention was 23.52. For reintervention alone, this number was 59.88. In the subgroup analysis of open and endovascular surgery, the NNH in open surgery patients for overall aortic events and reintervention was 54.72, compared to endovascular surgery, where it came out as 8.21 (see Calculation in the Supplemental Material).

## DISCUSSION

Our study’s most essential findings can be summarized as follows: (I) Due to the high number of late aortic events, it seems unreasonable to skip the first follow-up visit after 6 months in patients undergoing elective open surgical or endovascular aortic treatment. (II) Skipping the first visit would lead to a significant number of missed aortic events. However, (III) the monetary burden and the radiation exposure of a close follow-up interval need to be weighed against the benefit of an early preventative aortic intervention and warrant further research.

### Descriptive results

Our data are in line with current guidelines and reflect current practice well. In fact, younger patients and patients with root or ascending pathologies were treated by open surgery more often, while older patients, particularly with descending aortic pathologies, were treated endovascularly [7]. Postoperative outcome is adequately explained by these different treatment allocations. However, as our aim was not to infer causation for higher rates of outcomes based on surgical technique, but rather to evaluate current practice, these differences were not relevant to our following analyses.

Generally, patients who received open repair of the aorta had to stay in the hospital as well as the intensive care unit longer than their endovascular counterpart. This is one major proposed advantages of endovascular repair. This was of course expected in this cohort. It is important to remind ourselves at this point that it was at no point the aim of this study to compare open and endovascular surgery with each other, but rather to analyse postoperative trends to evaluate follow-up protocols for each of these subgroups [12].

#### Kaplan–Meier of open and endovascular surgery

Patients have a higher rate of aortic events and reinterventions during follow-up after receiving endovascular repair of the aorta. These findings are also present in current published data. In a meta-analysis by Giannopoulos *et al.*, the risk ratio of reintervention in open and endovascular abdominal aortic surgery was calculated to be around 2.18 with a confidence interval as high as 3.17 [13]. This coincides with our data, which propose about 3 times higher rates of reintervention after endovascular surgery compared to open surgery. Due to the definition of our study cohort, perioperative mortality was excluded from these analyses.

Another glaring difference in outcomes between open and endovascular surgery is regarding diameter progression. Diameter progression in endovascular techniques may happen through different mechanisms, and at different locations such as proximal and distal landing zones and at the excluded aneurysm sac. In open repair, this may only happen proximal and distal to the anastomoses. Additionally, open repair may act as a sort of scaffolding, stabilizing the surrounding aorta. Conversely, oversizing and consequently radial forces of endovascular prostheses might increase further aortic dilation [14].

These worse outcomes in patients following endovascular treatment during follow-up are most likely caused by a combination of previously mentioned older patients [12] as well as an inherent risk of endovascular surgery caused by the endovascular character itself. In open surgery, the diseased segment of the aorta is generally removed or excluded by permanent sutures, reducing the different possibilities for aortic events during follow-up. In endovascular surgery, as relevant segments of the aorta are only covered and bypassed by the stent graft, continued progression of aortic disease might still lead to problems down the line [7].

#### Kaplan–Meier of thoracic and abdominal surgery

Our data are generally comparable to published literature, except for marginally better outcome rates. This is most likely explainable by the fact that most published data are not limited to elective, non-emergent surgery, making a direct comparison difficult [15, 16]. There are two glaring differences in thoracic vs abdominal surgery: endoleaks and diameter progression.

Published data also suggest a difference in endoleak frequency. This observed difference might stem from a decrease in tunica media thickness and elastin content in more distal sections of the aorta, which may leave the aorta more prone to lasting dilation [17], which predisposes to type I endoleak. Consequently, the same mechanism would also be causative of the increased rate if diameter progression. Probably due to these higher rates of endoleak, as well as diameter progression, which are both included as at least IIb recommendations for reintervention in current guidelines [7], overall reintervention rates are higher in abdominal surgery.

#### Markov Chain Model

Regarding our simulation, it is suggested that by 6 months after discharge, 4.25% of patients who received either endovascular or open surgery for an aortic pathology will develop aortic events, 39.29% of which is an indication for reintervention. Now, assuming all the patients with an indication for reintervention would succumb to their complication before the 1-year follow-up, omitting the 6-month follow-up would lead to 1.67% (reinterventions) absolute increase in the risk of death, resulting in a NNH of 60 patients.

The same analysis for aortic events or indications for reinterventions in open and endovascular surgery subgroups found that as little as less than 10 patients who received endovascular treatment of aortic disease would need to skip the first follow-up visit after 6 months for one patient with a relevant outcome to be missed. After open surgery, this number rises to 58 patients. This difference is reflected in the current European Association for Cardio-Thoracic Surgery/Society of Thoracic Surgeons (EACTS/STS) aortic guidelines, which suggest different surveillance intervals after different kinds of interventions. For example, follow-up after endovascular aortic repair is generally recommended after 1 month, 6 months, 12 months and then annually thereafter [6, 7]. After open repair, if no residual aortopathy is left, there are some recommendations for more lenient follow-up algorithms, for example, after 1 year, and every 5 years after that [3]. This seems rather reasonable given our results. Especially in patients with known or ne kidney disease, who would potentially be harmed by frequent contrast agent exposure, longer follow-up intervals may be beneficial. Overall, the correct follow-up interval is a patient-specific decision formed data from studies such as this one, as well as patient-specific factors such as comorbidities or even distance to nearest hospital. In newer approaches to guiding surveillance intervals, patient-specific follow-up times have become more common. If after 1-month of follow-up, imaging does not give cause for concern, the 6-month follow-up visit may be skipped. The most recent guidelines for the treatment of aortic diseases recommend the first follow-up visit to be after 6 months [7]. However, as follow-up visits come with risks, it is important to remember that more does not always equal better. While aortic events such as diameter progression are detectable using ultrasound, during follow-up, the method of choice for finding others such as endoleaks is still computed tomography (CT) [7]. The high cumulative dose received during a life of follow-up CT imaging leaves patients at risk of newly developing solid organ malignancies [18].

Current recommendations for postoperative follow-up intervals are not based on published data addressing this specific issue. These recommendations were mainly formed by expert opinion based on clinical experience and in conjunction with published incidence rates found in observational data and trials conducted for a different reason [3, 7]. Future recommendations must weigh the increased risk caused by radiation during follow-up against the risk of missed postoperative complications to define optimal surveillance protocols, tailored to each indication and technique.

It should be noted that this might be due to limited established methodology directed at this matter. Therefore, it is difficult to compare our data to available literature, as it is the first of its kind. Moreover, no consideration for the acuity of the surgery has been made regarding variable postoperative surveillance protocols. In acute aortic disease, an argument might be made for more frequent postoperative follow-up. However, to support this notion, more data still need to be generated. As for our choice of methodology, a standard Markov Chain Model being time homogeneous would not be able to accurately depict our issue at hand, this is the reason why we opted for a discrete time non-homogenous model, with dynamic transition probabilities over time. As we cannot accurately assume factors influencing our outcomes, we chose to base our model on the most reliable metric available: outcome measured over time. As these could simply be used to calculate the transition probability matrices, a discrete time non-homogeneous model was used. In our model, we decided to use the discrete-time non-homogeneous approach with a 6-month interval due to two reasons. First, too short of a time-step interval might have led to overfitting in our model, which would have sacrificed the generalizability for the sake of improved temporal resolution [19]. We decided on 6 months specifically, as current guidelines generally suggest the first follow-up visit after discharge to happen around that time [7]. A non-homogeneous model was chosen, as it was evident by the Kaplan–Meier curves that the transition probabilities do not stay constant over time; therefore, to more accurately depict and simulate reality, we calculated specific probabilities for each time step.

### Limitations

Our work has two significant limitations to consider when applying its results. First, this study was performed retrospectively. Second, increased rates of patient censoring render the interpretation of data on long-term surveillance less beneficial at 1.5 years and thereafter. Lastly, in this analysis, we looked at common elective aortic surgery as a whole. Additional analyses of subgroups should be performed in future work. It is important to note, however, that this analysis and simulation is the first of its kind in patients after aortic surgery. Data on patients after elective aortic surgery are especially rare. To conclusively evaluate postoperative follow-up intervals, they first need to be precisely evaluated in observational studies such as this one, and then based on the results, prospective, randomized trials need to be conducted.

## CONCLUSION

It seems unreasonable to skip the first follow-up after 6 months, even in elective patients with uncomplicated postoperative course. Skipping the first visit would lead to a significant number of missed aortic events. The monetary burden and the radiation exposure of a close follow-up interval need to be weighed against the benefit of an early preventative aortic intervention and warrant further research.

## Supplementary Material

ivae226_Supplementary_Data

## Data Availability

Due to restrictions put forth by our ethics committee, raw data of this analysis may not distributed.

## Notes

IRB: 04/02/2021 (No. 20-1302)

## ABBREVIATIONS

CT: Computed tomography
MCM: Discrete time non-homogeneous Markov Chain Model
NNH: NUMBER needed to harm
PAU: Penetrating aortic ulcer
TEVAR: Thoracic endovascular aortic repair
