# Supplementary material for: Are current follow-up intervals justified in patients with non-emergent aortic surgeries?
Source: Interdiscip Cardiovasc Thorac Surg. 2024 Dec 28;40(1):ivae226. doi: 10.1093/icvts/ivae226 (PMC11706529; doi:10.1093/icvts/ivae226)
Supplement: ivae226_Supplementary_Data [file ivae226_supplementary_data.docx]

## Statistical analysis

**Kaplan-Meier:** We subsequently conducted analogous analyses on data derived from patients who exclusively underwent either open or endovascular surgery, as well as those who exclusively underwent Thoracic or Abdominal surgery. We then also stratified some of the Kaplan-Meier curves into original pathologies, namely aneurysm and PAU. Comparisons were performed using the log-rank test.

**Markov-Chain:** The different states used were healthy, complication, reintervention, and death. Patients could transition from a healthy state directly into a state of complication, reintervention, or death; from a state of complication into a state of reintervention or death; from a state of reintervention into a state of death; death was an absorbing state. The Markov chain model used in this work was visualized in **Figure 2**. Illustrations of the Markov-chain model with respective transition probabilities at each timepoint can be found in the supplemental materials (**Tables S1-S6**). Risks were then calculated based on this model and an estimate of a Number Needed to Harm ( NNH ) was calculated to possibly predict the consequences of skipping the first follow-up visit after 6 months.

The following figures depict the discrete time non-homogeneous markov-chain model at each timpoint up to 3 years of follow-up.

| 0 - 6 months | |  |  |  |
| --- | --- | --- | --- | --- |
|  | Healthy | Complication | Reintervention | Death |
| Healthy | 0.9525852 | 0.02586207 | 0.01724138 | 0.004310345 |
| Complication | / | / | / | / |
| Reintervention | / | / | / | / |
| Death | / | / | / | / |

Table S1.: Transition propabilities for the given time-span.

| 6 - 12 months | |  |  |  |
| --- | --- | --- | --- | --- |
|  | Healthy | Complication | Reintervention | Death |
| Healthy | 0.938914 | 0.03393665 | 0.02031699 | 0.00678733 |
| Complication | 0 | 1 | 0 | 0 |
| Reintervention | 0 | 0 | 1 | 0 |
| Death | 0 | 0 | 0 | 1 |

Table S2.: Transition propabilities for the given time-span.

| 12 - 18 months | |  |  |  |
| --- | --- | --- | --- | --- |
|  | Healthy | Complication | Reintervention | Death |
| Healthy | 0.9807229 | 0.007228916 | 0.009638554 | 0.002409639 |
| Complication | 0 | 0.962962963 | 0 | 0.037037037 |
| Reintervention | 0 | 0 | 1 | 0 |
| Death | 0 | 0 | 0 | 1 |

Table S3.: Transition propabilities for the given time-span.

| 18 - 24 months | |  |  |  |
| --- | --- | --- | --- | --- |
|  | Healthy | Complication | Reintervention | Death |
| Healthy | 0.972973 | 0.00982801 | 0.007371007 | 0.00982801 |
| Complication | 0 | 0.96551724 | 0.034482759 | 0 |
| Reintervention | 0 | 0 | 1 | 0 |
| Death | 0 | 0 | 0 | 1 |

Table S4.: Transition propabilities for the given time-span.

| 24 - 30 months | |  |  |  |
| --- | --- | --- | --- | --- |
|  | Healthy | Complication | Reintervention | Death |
| Healthy | 0.979798 | 0.002525253 | 0.007575758 | 0.01010101 |
| Complication | 0 | 0.9375 | 0 | 0.0625 |
| Reintervention | 0 | 0 | 0.96 | 0.04 |
| Death | 0 | 0 | 0 | 1 |

Table S5.: Transition propabilities for the given time-span.

| 30 - 36 months | |  |  |  |
| --- | --- | --- | --- | --- |
|  | Healthy | Complication | Reintervention | Death |
| Healthy | 0.9896907 | 0 | 0 | 0.01030928 |
| Complication | 0 | 0.9677419 | 0 | 03225806 |
| Reintervention | 0 | 0 | 1 | 0 |
| Death | 0 | 0 | 0 | 1 |

Table S6.: Transition propabilities for the given time-span.

## Patient baseline characteristics

There were higher rates of previous cardiovascular surgery (66 (57%) vs 83 (24%); p-value < 0.001) in patients who underwent endovascular surgery. In contrast, more patients with open surgery had a bicuspid aortic valve (0 (0%) vs 79 (23%); p-value < 0.001). Considering the causal pathology for aortic treatment, patients undergoing open surgery had a higher rate of aneurysm (94 (82%) vs 326 (93%); p-value < 0.001), and a lower rate of PAU (25 (22%) vs 9 (2.6%); p-value < 0.001). Two hundred-nine (45%) patients were treated for abdominal aortic pathologies.

|  |  | **Surgery** | |
| --- | --- | --- | --- |
| **Characteristic** | **Overall**, N = 464*^1^* | **Endovascular**, N = 116*^1^* | **Open**, N = 348*^1^* |
| Valve-bearing conduit | 37 (8.0%) | 0 (0%) | 37 (11%) |
| Valve sparing root replacement | 79 (17%) | 0 (0%) | 79 (23%) |
| Ascending aorta replacement | 69 (15%) | 0 (0%) | 69 (20%) |
| Hemiarch replacement | 64 (14%) | 0 (0%) | 64 (18%) |
| Total arch replacement | 18 (3.9%) | 0 (0%) | 18 (5.2%) |
| Frozen elephant trunk | 11 (2.4%) | 0 (0%) | 11 (3.2%) |
| Aortic valve surgery | 66 (14%) | 0 (0%) | 66 (19%) |
| Mitral valve surgery | 12 (2.6%) | 0 (0%) | 12 (3.4%) |
| Tricuspid valve surgery | 10 (2.2%) | 0 (0%) | 10 (2.9%) |
| Coronary Bypass Surgery | 28 (6.0%) | 0 (0%) | 28 (8.0%) |
| Open abdominal aortic aneurysm | 110 (24%) | 0 (0%) | 110 (32%) |
| Abdominal endovascular aortic repair | 93 (20%) | 93 (80%) | 0 (0%) |
| Carotico-Subclavian bypass | 13 (2.8%) | 13 (11%) | 0 (0%) |
| Thoracic endovascular aortic repair | 22 (4.7%) | 22 (20%) | 0 (0%) |
| *^1^*n (%) | | | |

Table S7.: Operative Details of the patient cohort.

|  |  | **Surgery** | |  |
| --- | --- | --- | --- | --- |
| **Characteristic** | **Overall**, N = 464*^1^* | **Endovascular**, N = 116*^1^* | **Open**, N = 348*^1^* | **p-value***^2^* |
| Follow-up (years) | 2.42 (1.32, 3.32) | 2.84 (1.17, 3.56) | 2.27 (1.35, 3.22) | 0.042 |
| Days in the ICU | 2.00 (1.00, 3.00) | 1.00 (1.00, 2.00) | 2.00 (1.00, 4.00) | <0.001 |
| Days in the hospital overall | 8.00 (5.00, 13.00) | 7.00 (2.00, 9.00) | 9.50 (6.00, 14.00) | <0.001 |
| Aortic events during Follow-up | 52 (11%) | 38 (33%) | 14 (4.0%) | <0.001 |
| Reintervention during Follow-up | 33 (7.1%) | 13 (11%) | 20 (5.7%) | 0.048 |
| Death during Follow-up | 23 (5.0%) | 12 (10%) | 11 (3.2%) | 0.002 |
| Endoleak during Follow-up | 32 (6.9%) | 32 (28%) | 0 (0%) | <0.001 |
| Anastomotic aneurysm during Follow-up | 19 (4.1%) | 7 (6.0%) | 12 (3.4%) | 0.3 |
| Aneurysm progression during Follow-up | 13 (2.8%) | 11 (9.5%) | 2 (0.6%) | <0.001 |
| *^1^*Median (IQR); n (%) | | | | |
| *^2^*Wilcoxon rank sum test; Pearson's Chi-squared test; Fisher's exact test | | | | |

Table S8.: Postoperative outcomes during Follow-up.

## Results

The following figures depict the comparison between different strata of patients in regard to the outcomes specified in our methods and materials.


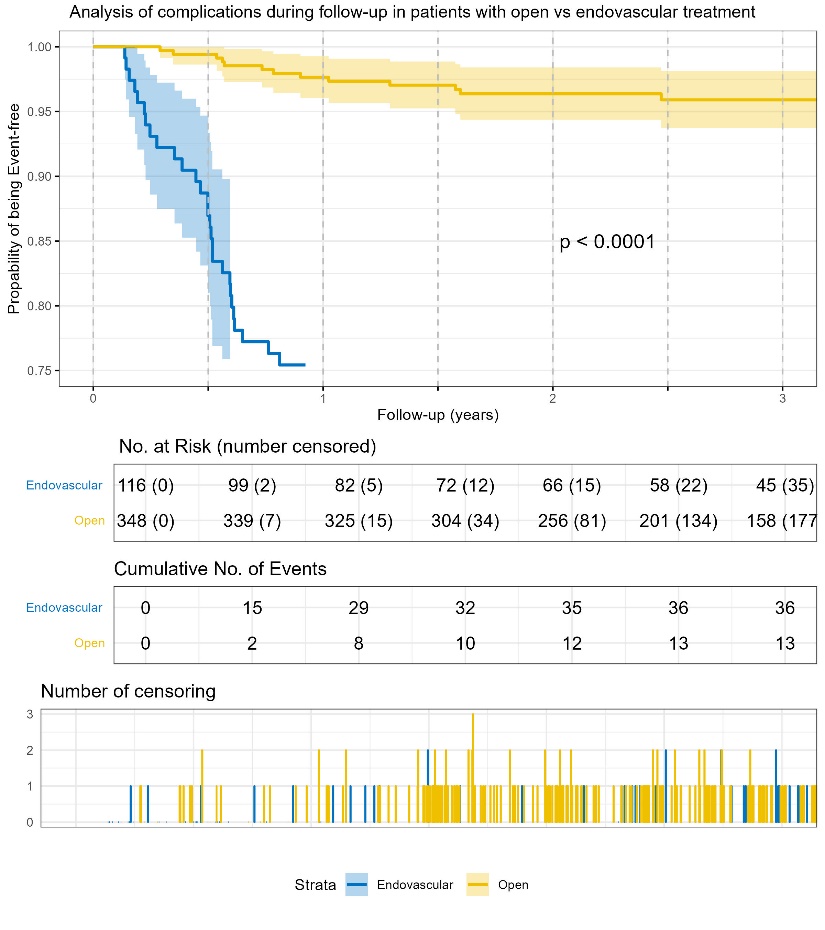


Figure S1.: Kaplan-Meier analysis and risk table including cumulative number at risk, number of censored patients, and number of events; Number of censoring illustrated as bar-chart over time.


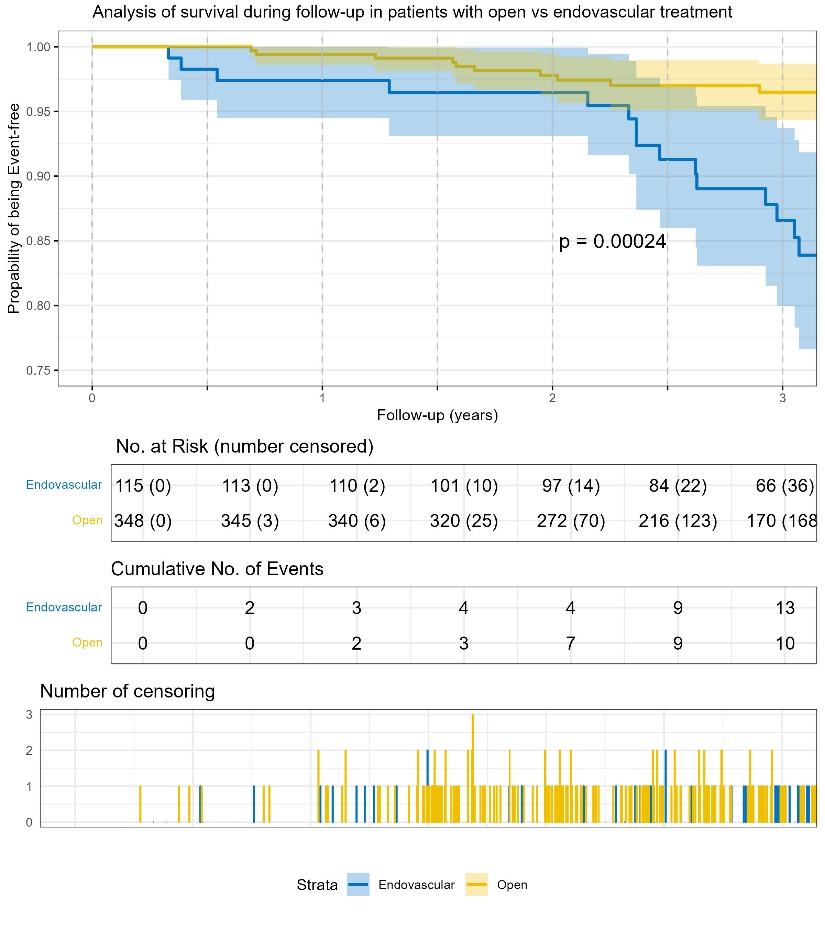


Figure S2.: Kaplan-Meier analysis and risk table including cumulative number at risk, number of censored patients, and number of events; Number of censoring illustrated as bar-chart over time.


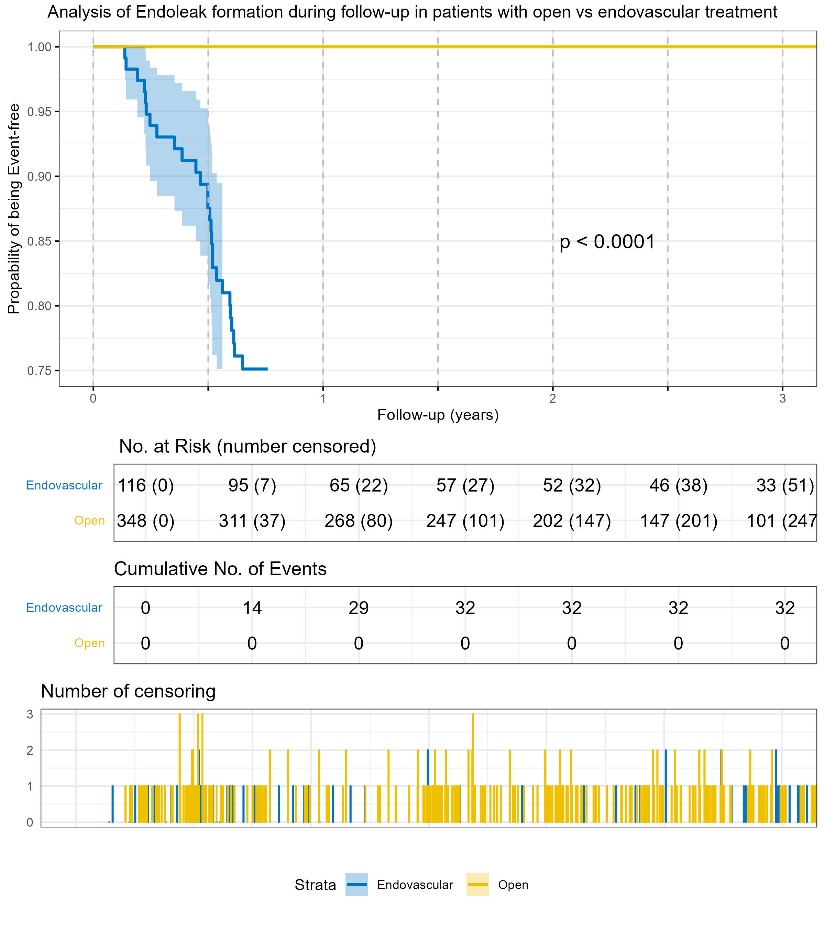


Figure S3.: Kaplan-Meier analysis and risk table including cumulative number at risk, number of censored patients, and number of events; Number of censoring illustrated as bar-chart over time.


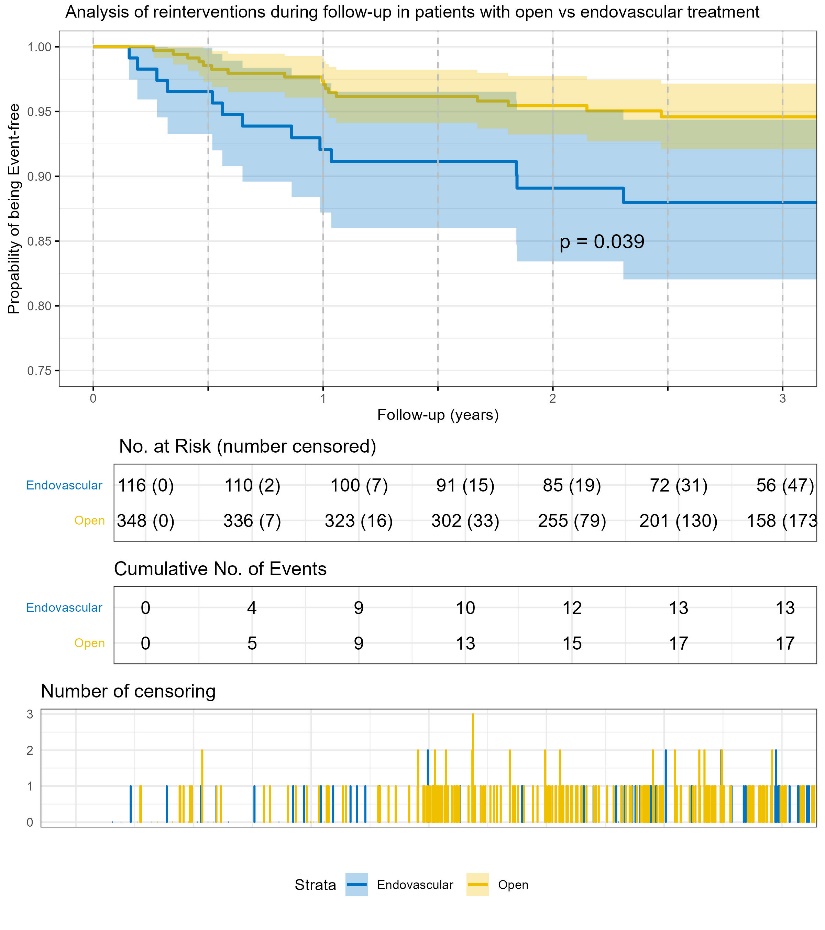


Figure S4.: Kaplan-Meier analysis and risk table including cumulative number at risk, number of censored patients, and number of events; Number of censoring illustrated as bar-chart over time.


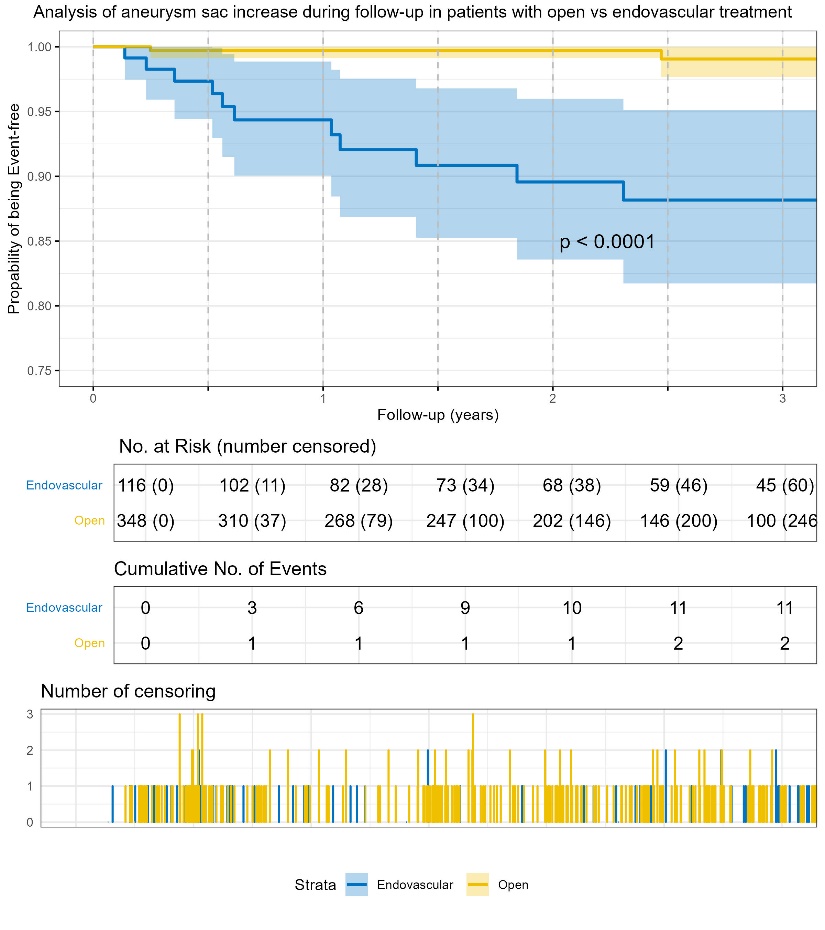


Figure S5.: Kaplan-Meier analysis and risk table including cumulative number at risk, number of censored patients, and number of events; Number of censoring illustrated as bar-chart over time.


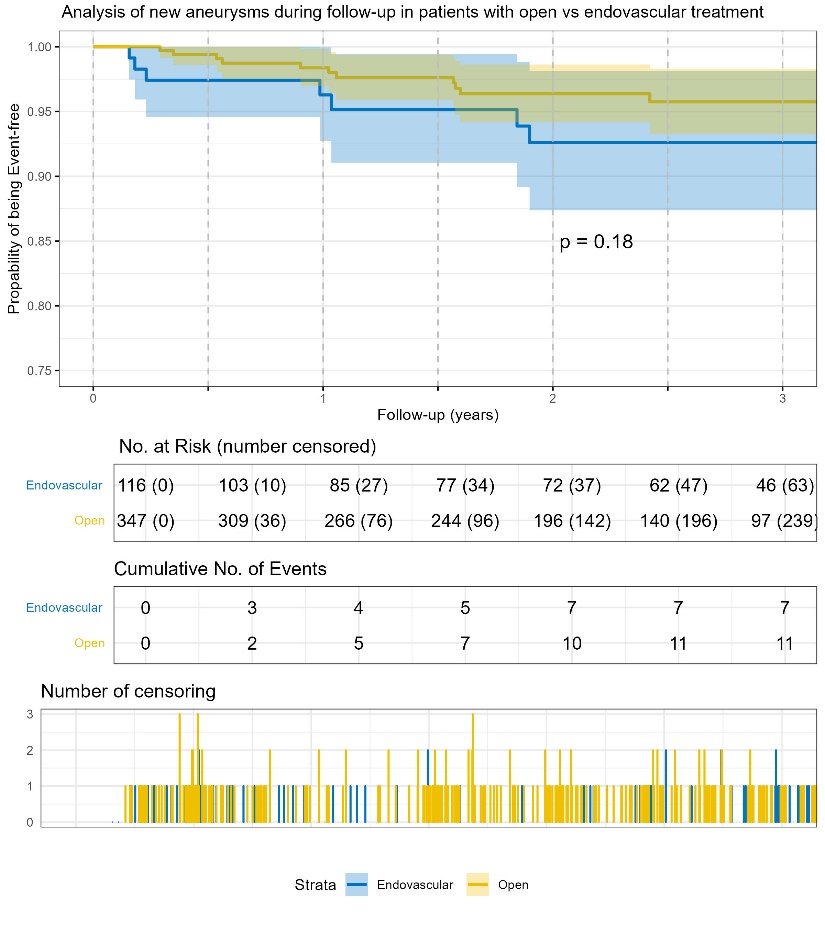


Figure S6.: Kaplan-Meier analysis and risk table including cumulative number at risk, number of censored patients, and number of events; Number of censoring illustrated as bar-chart over time.


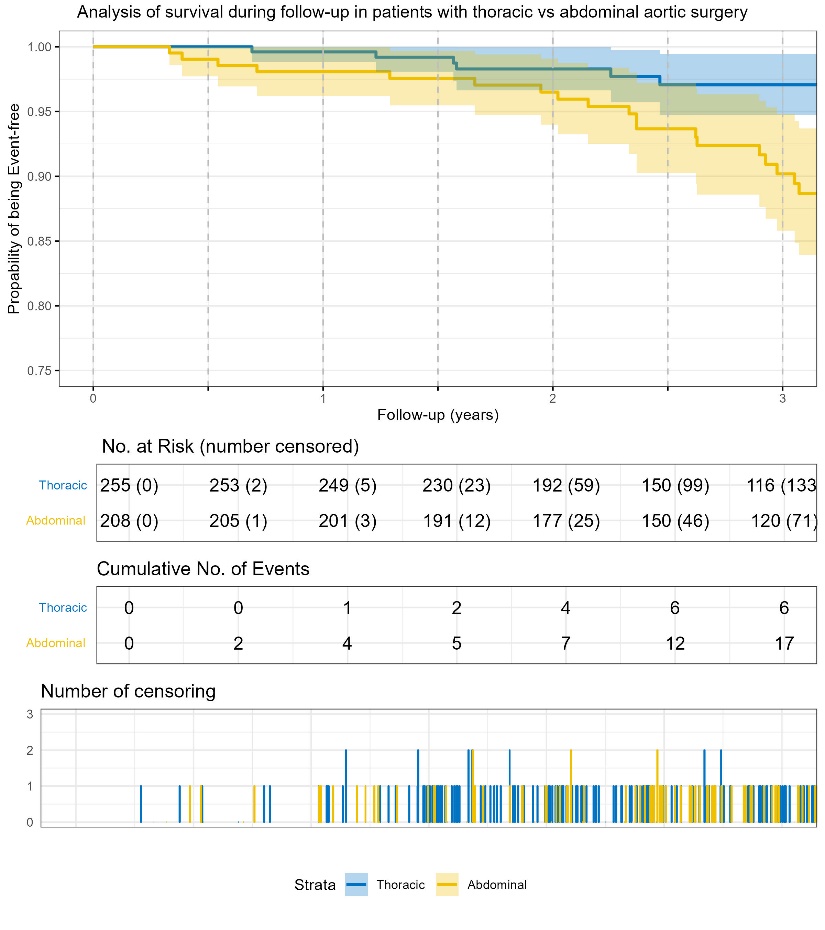


Figure S7.: Kaplan-Meier analysis and risk table including cumulative number at risk, number of censored patients, and number of events; Number of censoring illustrated as bar-chart over time.


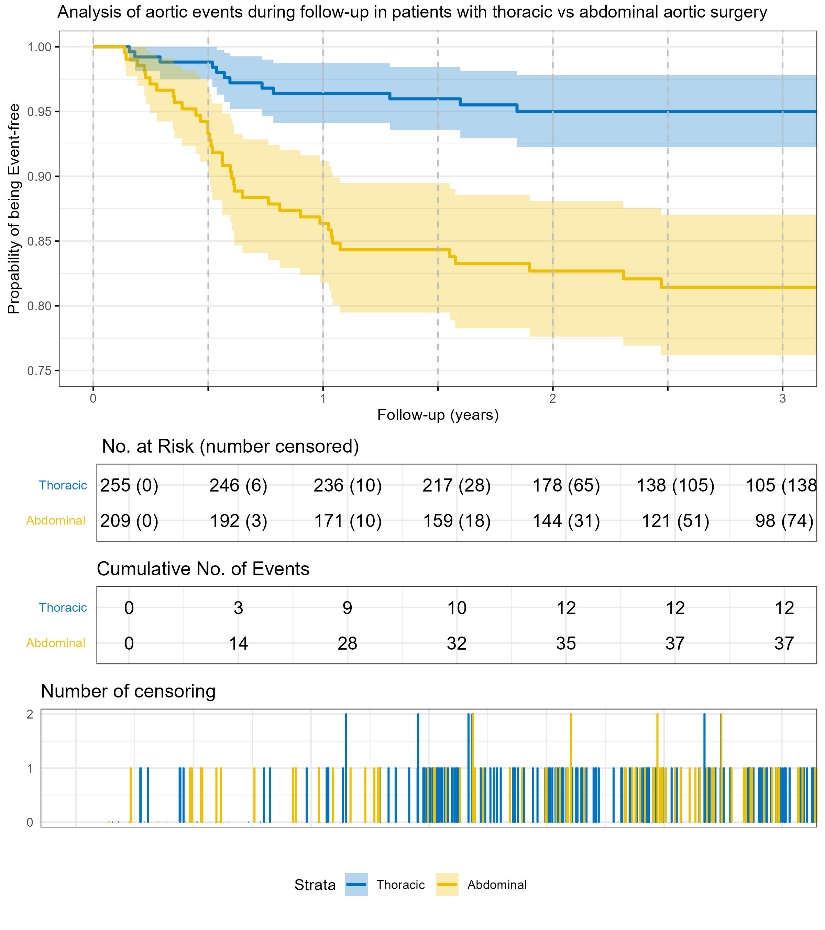


Figure S8.: Kaplan-Meier analysis and risk table including cumulative number at risk, number of censored patients, and number of events; Number of censoring illustrated as bar-chart over time.


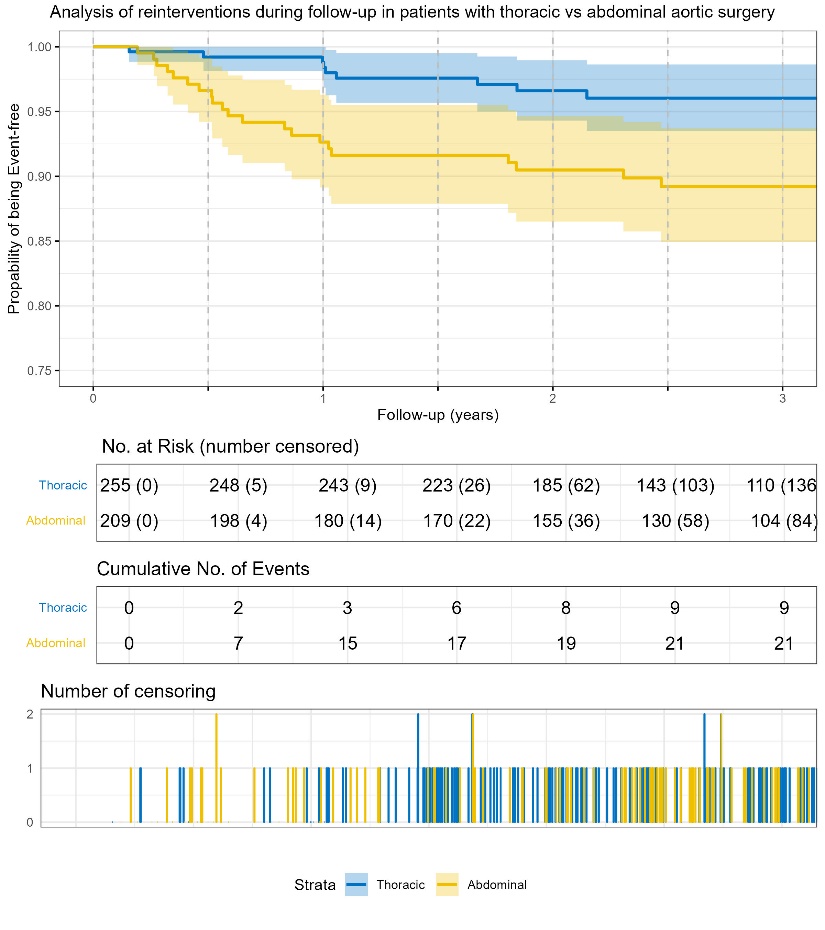


Figure S9.: Kaplan-Meier analysis and risk table including cumulative number at risk, number of censored patients, and number of events; Number of censoring illustrated as bar-chart over time.


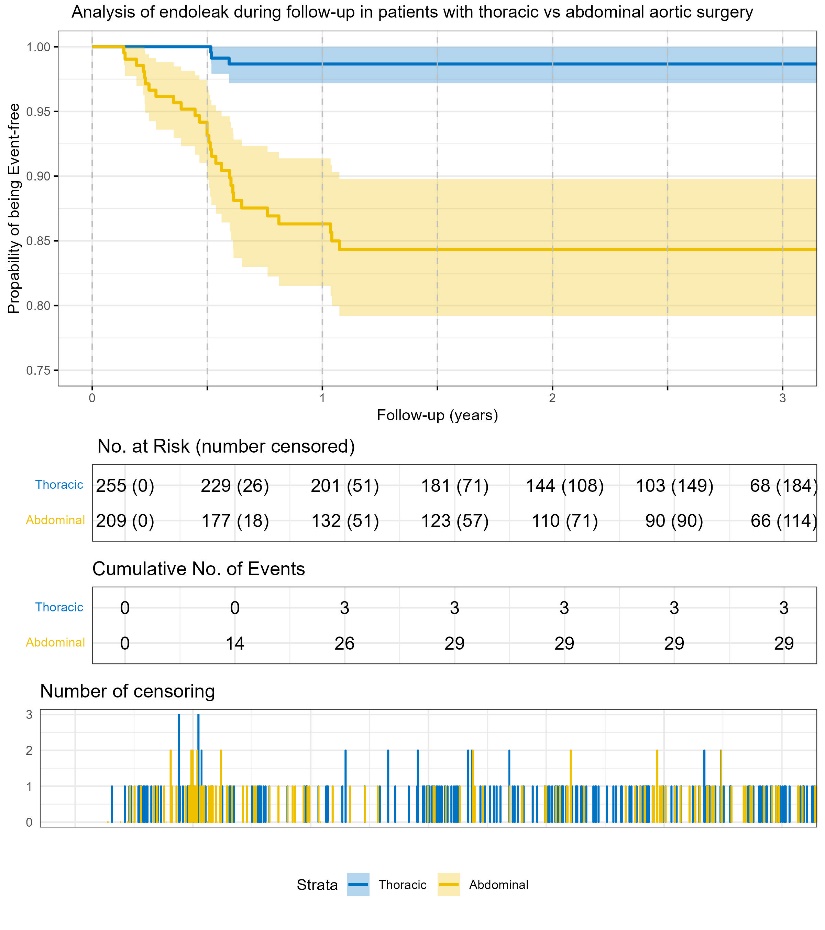


Figure S10.: Kaplan-Meier analysis and risk table including cumulative number at risk, number of censored patients, and number of events; Number of censoring illustrated as bar-chart over time.


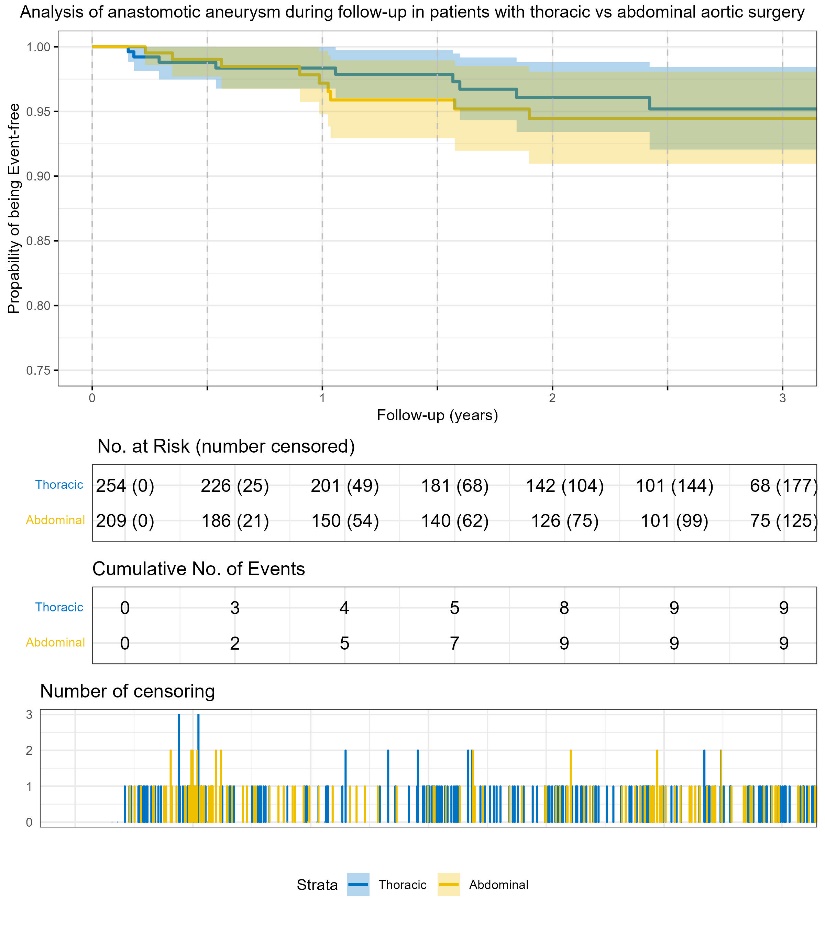


Figure S11.: Kaplan-Meier analysis and risk table including cumulative number at risk, number of censored patients, and number of events; Number of censoring illustrated as bar-chart over time.


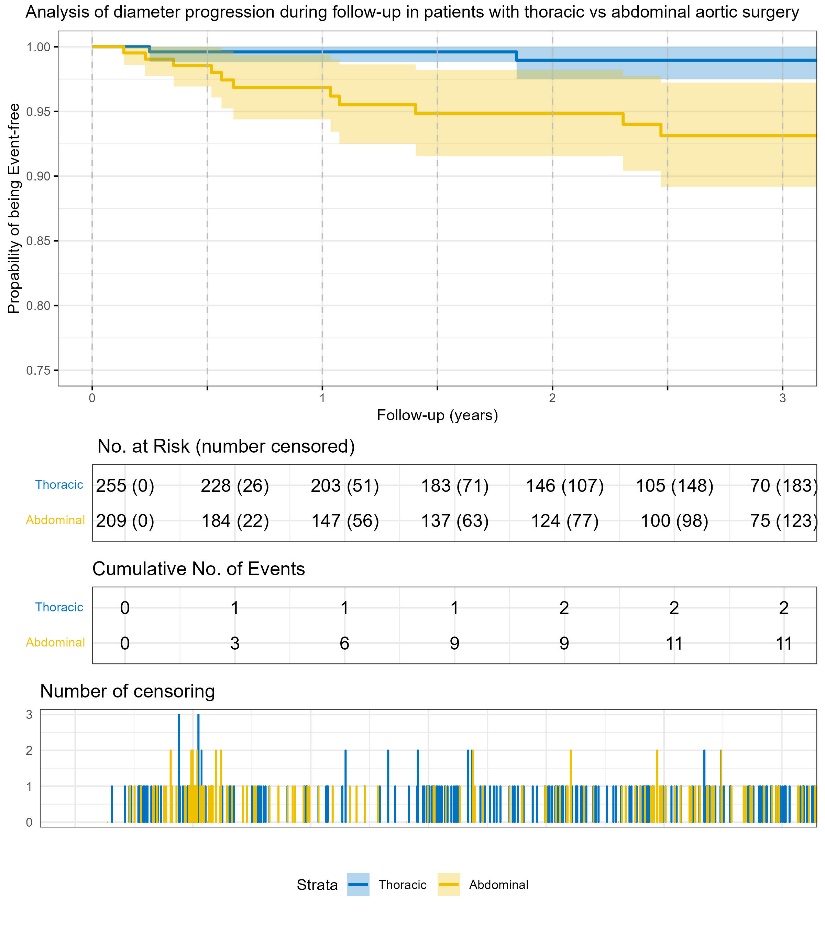


Figure S12.: Kaplan-Meier analysis and risk table including cumulative number at risk, number of censored patients, and number of events; Number of censoring illustrated as bar-chart over time.

## Discussion

Number needed to Harm (NNH):

Assumption: All Patients who would have needed to receive a reintervention would have died

NNH = 1/0.0167 (Absolute risk increase for death) = 59.88024

NNH = 1/0.0425 (absolute risk increase overall) = 23.52941

NNH for Open surgery: 54.72431

NNH for Endovascular surgery: 8.21349
